# Unveiling the knowledge domain and emerging trends of olfactory dysfunction with depression or anxiety: A bibliometrics study

**DOI:** 10.3389/fnins.2022.959936

**Published:** 2022-09-08

**Authors:** Fangwei Zhou, Tian Zhang, Ying Jin, Yifei Ma, Yangsong Li, Mengting Zeng, Guodong Yu

**Affiliations:** Department of Otorhinolaryngology Head and Neck Surgery, Affiliated Hospital of Guizhou Medical University, Guiyang, China

**Keywords:** olfactory dysfunction, depression, anxiety, visual analysis, bibliometric analysis

## Abstract

Olfactory dysfunction (OD) accompanied by depression or anxiety is a very common clinical problem, and there has been a growing number of studies on OD with depression or anxiety in recent decades. This study performed bibliometric and visual analyses of the literature on OD with depression or anxiety to derive research trends and identify emerging research foci. Relevant publications were obtained from the Science Citation Index-Expanded and Social Sciences Citation Index in the Web of Science Core Collection databases (2002–2021). CiteSpace and VOSviewer were applied to identify and evaluate research foci and emerging trends in this research domain. The analyses found that the number of publications related to OD with depression or anxiety has increased significantly over the past 20 years, up from 15 in 2002 to 114 in 2022. The country that ranked highest in the number of articles and international cooperation was the United States. The top 10 most frequent keywords were “depression,” “olfaction,” “anxiety,” “dysfunction,” “olfactory bulbectomy,” “olfactory dysfunction,” “Parkinson’s disease,” “odor identification,” “brain,” and “disorders.” Analysis of keywords with the strongest citation bursts revealed that “oxidative stress” is an emerging research hotspot. A timeline chart of the cluster of co-cited references demonstrated that Parkinson’s disease was always a topic of interest in this area of research. This study conducted an objective, comprehensive, and systematic analysis of these publications, and identified the development of trends and hotspots in this research domain. It is hoped that this work will provide scholars, worldwide, with information to assist them in further research and the development of new therapies.

## Introduction

The sense of smell is one of the five human senses and it affects almost every aspect of life, including the creation of new life, mate selection, hygiene, diet, and the perception of danger from gas and smoke (Croy et al., 2014). It has been estimated that 3–20% of the worldwide population suffers from olfactory dysfunction (OD) (Marin et al., 2018). Approximately one-third of patients with OD experience severe suffering and have a marked decrease in quality of life (Croy et al., 2014; Marin et al., 2018). Epidemiological studies have found that the prevalence and severity of OD increase with age, but the underlying mechanism is unclear (Ottaviano et al., 2013; Attems et al., 2015). Common causes of OD are upper respiratory infections, head trauma, and sinus disease, which account for about two-thirds of all OD cases (Ciofalo et al., 2006). In addition, environmental pollution, drugs, toxic chemicals, and nutritional disorders can also lead to OD (Murphy et al., 2002). Tobacco smoking can lead to a reversible decrease in the sense of smell, whereas chronic rhinosinusitis with nasal polyps may cause partial or complete absence of smell (Vennemann et al., 2008; Guilemany et al., 2011). Recent research indicates that there is an association between OD and neurodegenerative diseases, and the high prevalence of OD, its early presentation, its persistence throughout the disease process, and the simplicity of olfactory testing have created interest in investigating OD as an early indicator of neurodegenerative disorders, such as Alzheimer’s disease, Parkinson’s disease (PD), and Lewy body dementia (Marin et al., 2018). Employing smell as a biomarker of neurodegenerative disorders is useful for prophase characterization, early diagnoses, differential diagnoses, and predicting the clinical outcomes of neurodegenerative disorders (Fullard et al., 2017).

It is well known that OD can affect the physical and mental health of people. Individuals with OD are more likely to suffer from anxiety, depression, and even have a dramatically greater risk of death, because of their inability to respond on time to dangerous odors, such as leaking gas, toxic chemicals, and rotting food (Hummel et al., 2016). The olfactory bulbs are considered the first processing centers of olfaction and they assist in the detection and recognition of odors (Smith and Bhatnagar, 2019). Studies suggest that the olfactory bulbs and their central connections probably have a crucial function in emotional behavior (Liu et al., 2021), and research has demonstrated that removing the olfactory bulbs induces depression-like behavior in animals after a “latency period” (Yuan and Slotnick, 2014). This latency period might represent the time required for follow-up changes in the limbic and hypothalamic bulbar targets that have an essential role in regulating emotion and memory. Depression-like behavior in animals following the removal of the olfactory bulbs is associated with changes in neurotransmission and endocrine and immune responses that have similarities to those observed in humans with depression (Song and Leonard, 2005). In the past few decades, researchers throughout the world have conducted studies in understanding the epidemiology, mechanisms, and association of OD with anxiety and depression. However, there is a lack of comprehensive reports that can help researchers obtain a visual overview of research trends in this field.

Bibliometrics is the utilization of mathematical-statistical approaches to summarize scientific activity in a field of research and identify emerging frontiers and foci, or increasing patterns of research, using literature databases and metrological features (Ma et al., 2021). Since its birth in the 1920s, bibliometrics has given birth to evaluation metrics such as journal impact factors, citations, and Altmetrics, as well as bibliometric methods such as citation analysis and scientific knowledge mapping (Fanelli and Glänzel, 2013; Bornmann and Marx, 2016). After the 1960s, with the rise of complex network analysis, bibliometrics began to expand from unidimensional statistical analysis to network analysis based on binary co-occurrence relationships, such as bibliographic coupling, co-citation analysis, co-authorship network analysis, author co-citation analysis, and journal co-citation analysis (Waltman et al., 2014). In short, bibliometrics is a discipline that studies the distribution structure, quantitative relationships, and change patterns of documentary intelligence. Compared to conventional review articles, the bibliometric analysis offers an up-to-date, intuitive, and non-biased approach to identifying trends and exploring specific domains of expertise, presented in the form of visual maps (Zhu and Zhang, 2021). Importantly, bibliometrics can reveal different aspects of statistical rules, patterns of association, and evolutionary dynamics in the literature collections and the research fields they represent. Besides describing and predicting trends in a domain, bibliometric analysis can also be used to compare contributions made by different countries/regions, institutions, authors, and journals (Tang et al., 2021). Several forms of visualization software, such as CiteSpace, VOSviewer, and HistCite, have been created to assist scholars in building knowledge maps, assessing the latest frontier advances in research, and visualizing trends in scientific publications (Wang and Meng, 2021). To date, those bibliometric tools have been applied to assess research trends and “hotspots” in various fields, such as disease treatment, pain management, toxicology, molecular biology, environmental pollution, food safety, urban hazards, and novel materials (Cheng et al., 2021; Mostafaie et al., 2021; Wu C. C. et al., 2021; Fu et al., 2022).

Although previous reviews have provided an outline for the theoretical and empirical elements of the relationship between OD and depression, to our knowledge, none of these articles have used quantitative or visualization methods to investigate the longitudinal and cross-sectional features, trends, and current hotspots in research on this topic. Thus, this study collected qualitative and quantitative data on publications and identified the most prolific or influential countries, institutions, authors, journals, and citations in this area. The purpose of this research was to conduct a thorough bibliometric analysis of research related to OD with depression or anxiety from 2002 to 2021, as well as to provide scholars who have already entered or will soon enter the field with new perspectives on the status of relevant studies, emerging trends, and future research directions, from a global perspective.

## Materials and methods

### Data collection and search strategy

**FIGURE 1:**
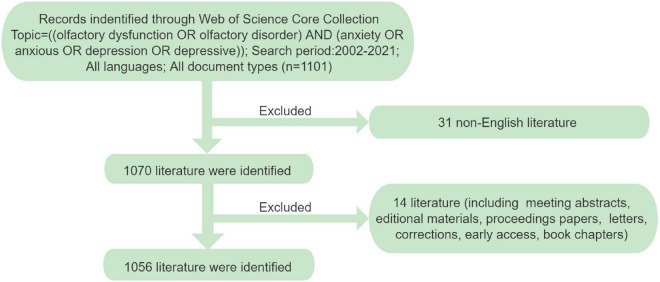
Flowchart of the literature selection process in this study.

This study performed a bibliometric analysis using the Science Citation Index-Expanded and Social Sciences Citation Index of the Web of Science Core Collection (WoSCC) database. The search phrase was: Topics = [(olfactory dysfunction OR olfactory disorder) AND (anxiety OR anxious OR depression OR depressive)]. The language of the documents was limited to English. We thoroughly searched the databases for relevant publications between 2002 and 2021, and only original articles and reviews were included in our analysis; meeting abstracts, editorial material, letters, and book chapters were excluded. A detailed flowchart of this study’s process for selecting articles is shown in Figure 1. To avoid bias from the continuous updates of databases, all publication searches and document downloads were conducted only on 25 May 2022. Two researchers in this study checked the data collection and entry independently. Differences in the results obtained by the two researchers were resolved by consensus through discussion or by consulting with experts in the field.

### Statistical analysis

The bibliometric analysis conducted in this study employed CiteSpace 5.8.R3 (Drexel University, Philadelphia, PA, United States), VOSviewer 1.6.16 (Leiden University, Leiden, Netherlands), and an online bibliometric analysis tool.^1^ Before conducting the bibliometric analysis, the downloaded documents were converted to plain text files, which were later uploaded to the bibliometric analysis tools. The processing of the data produced a range of maps of visual knowledge areas and identified emerging trends and landmark literature, which were classified by countries, institutions, authors, journals, keywords, and citations. In addition, their H-index, impact factor, and category quartile were determined based on the 2021 Journal Citation Report. A journal’s impact factor is an internationally recognized index for evaluating the impact of journals (Eyre-Walker and Stoletzki, 2013), and the H-index is regarded as a crucial parameter for determining the scientific influence of journals, authors, institutions, and countries/regions (Wu H. et al., 2021).

CiteSpace is a visualization analysis tool that concentrates on analyzing the underlying knowledge contained in scientific publications, which has evolved in the context of scientometrics and the visualization of data. The visual network diagrams of this study consist of many nodes and lines. The nodes represent various items, such as countries/regions, institutions, authors, and co-cited references. The size of a single node reflects the number of observed items. The thickness of each circle indicates the number of citations relative to the “time zone” or time frame, and the lines between nodes refer to the collaboration/co-occurrence/co-citation of articles. CiteSpace was also applied in this study to compute the centrality of countries, institutions, and authors. Centrality is used to evaluate the importance of a node in a visual network. The higher the number of links through a node in a network, the higher the centrality of that node.

VOSviewer software is commonly used to create bibliometric visualization diagrams. It can be used to create maps of authors, institutions, countries, or journals based on collaborative relationships, or to establish keyword networks based on their concurrence. In contrast to commonly used bibliometric programs, VOSviewer focuses particularly on bibliometric graphical representation, which is especially useful for visualizing large bibliographies in an easily interpretable manner. The major aim of using VOSviewer was to analyze bibliometric networks and construct visualization diagrams, ultimately achieving an in-depth and comprehensive understanding of the structure and growing trends in scientific research. VOSviewer software can build three types of visual maps: network maps, temporal overlay maps, and density visualization maps. In this study, VOSviewer was applied to evaluate keywords and research teams and to create density graphs.

## Results

### General information

**FIGURE 2:**
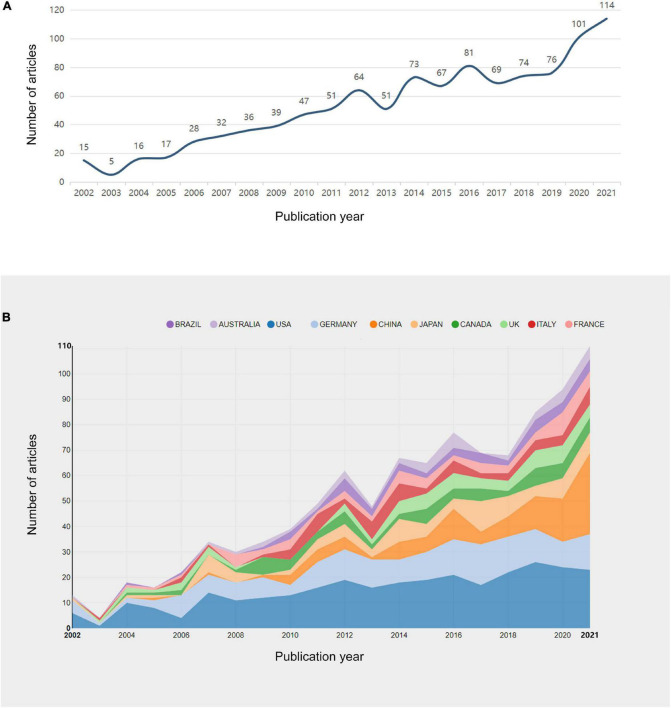
**(A)** Number of publications per year (2002–2021). **(B)** Top 10 productive countries in the field of OD with depression or anxiety (2002–2021).

A total of 1,056 publications (including 867 original articles and 189 review articles) met the inclusion criteria of being published between 2002 and 2021. The number of publications has grown significantly during the last 20 years, which suggests a growing interest in the association of OD with depression and anxiety (Figure 2A). Annual publications rose from 15 in 2002 to 114 in 2021. These 1,056 publications were cited 41,480 times (average per item = 39.28, H-index = 89). Research activity peaked in 2017–2021, with 434 papers published during this 5-year period, accounting for more than 40% of the total number of publications. Each original article or review was marked with at least one subject category in the WoSCC database. A total of 83 subject categories were assigned to the 1,056 publications about OD with depression or anxiety. The largest number of publications fell into the category of neuroscience (486), followed by psychiatry (216) and clinical neurology (208).

### Distribution by countries/regions and institutions

**FIGURE 3:**
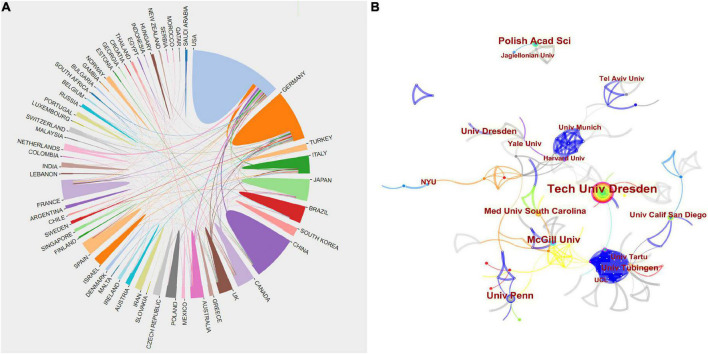
**(A)** Cooperation network of prolific countries/regions. **(B)** Visualization map of institutions’ cooperative relations for research on OD with depression or anxiety.

Researchers from 1,453 institutions in 61 countries/regions have published papers in this field. The top 10 countries and institutions that have contributed the most publications to research on this subject are shown in Tables 1, 2, respectively. The top five countries with the most articles are the United States (304), Germany (183), China (112), Japan (86), and Canada (64) (Figure 2B). Figure 3A, which maps cooperation networks among countries/regions, illustrates that the top five countries with the highest centrality scores were the United States (0.56), the United Kingdom (0.19), France (0.15), Italy (0.04), and Brazil (0.15). The CiteSpace analysis of the distribution of institutions contributing to the literature in the field (Figure 3B) revealed that the five most productive institutions were the Technische Universitat Dresden (62), Institut National de la Sante et de la Recherche (36), National Institutes of Health (26), Harvard University (25), and University of California (25). The top three institutions, ranked by their centrality, were all located in the United States: University of California (0.13), Harvard University (0.12), and National Institutes of Health (0.08).

**TABLE 1:** Top 10 most countries in published research on OD with depression or anxiety from 2002 to 2021.

Rank	Country	Output	Centrality
1	United States	304	0.56
2	Germany	183	0.12
3	China	112	0.03
4	Japan	86	0.04
5	Canada	64	0.00
6	Italy	59	0.15
7	France	56	0.15
8	United Kingdom	54	0.19
9	Brazil	45	0.15
10	Australia	40	0.00

**TABLE 2:** Top 10 institutions in research on OD with depression or anxiety from 2002 to 2021.

Rank	Institutions	Output	Centrality	Country
1	Technische Universitat Dresden	62	0.04	Germany
2	Institut National de la Sante et de la Recherche	36	0.00	France
3	National Institutes of Health	26	0.08	United States
4	Harvard University	25	0.12	United States
5	University of California	25	0.13	United States
6	Centre National de la Recherche Scientifique	23	0.00	France
7	Polish Academy of Sciences	23	0.00	Poland
8	University of London	22	0.00	United Kingdom
9	University of Pennsylvania	21	0.02	United States
10	McGill University	20	0.04	Canada

### Distribution by author

**FIGURE 4:**
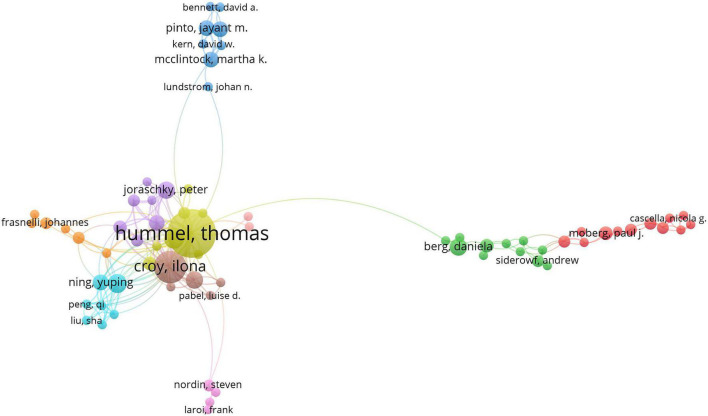
The international cooperation among relevant authors.

A large amount of research was generated by 5,422 authors. Table 3 shows the top 10 most productive authors, and Figure 4 shows the collaboration networks of authors. The distribution of collaborations shown in Figure 4 reveals that the relationship between these authors is quite fragmented. This means that the scale of collaboration among academics is relatively small and generally inadequate. The largest number of publications were authored by T. Hummel of Technische Universität Dresden (45 publications; 1,878 citations), followed by I. Croy of the Friedrich Schiller University of Jena (21 publications; 1,128 citations) and Z. M. Soler of the Medical University of South Carolina (11 publications; 419 citations), with T. Hummel having the highest H-index (21). None of the authors had a centrality score that reached 0.01, suggesting that there was a relatively low level of cooperation among academic teams from different institutions.

**TABLE 3:** Top 10 authors of articles on OD with depression or anxiety from 2002 to 2021.

Rank	Author	Output	Centrality	Total citations	Average citations	H-index
1	Hummel T.	45	0.00	2,196	48.80	21
2	Croy I.	21	0.00	1,128	53.71	13
3	Soler Z. M.	11	0.00	419	38.09	9
4	Atanasova B.	10	0.00	335	33.50	6
5	Schlosser R. J.	10	0.00	271	27.10	8
6	Shoenfeld Y.	10	0.00	460	46.00	10
7	Xie P.	9	0.00	213	23.67	8
8	Berg D.	8	0.00	549	68.63	7
9	Mahesh R.	8	0.00	239	29.88	7
10	Nowak G.	8	0.00	410	51.25	6

### Keywords analysis

**FIGURE 5:**
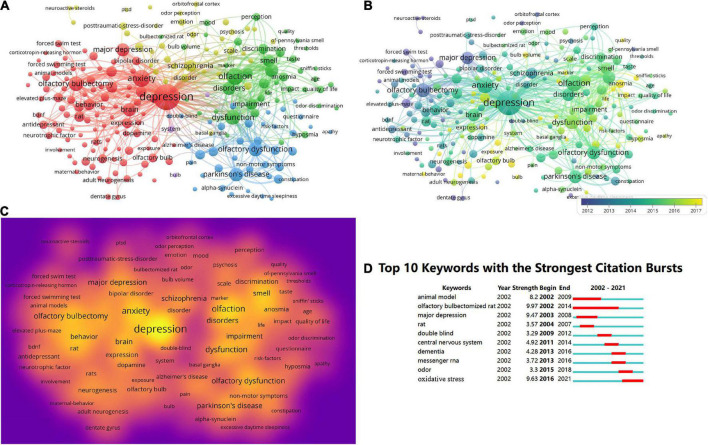
**(A)** Network visualization diagram of keyword co-occurrence analysis. **(B)** Distribution of keywords based on the temporal order of their co-occurrence. **(C)** Density network visualization graph of keywords. **(D)** Top 20 keywords with the strongest citation bursts.

Figure 5 shows the maps resulting from the VOSviewer analysis of the 1,056 publications. Figure 5A includes 221 terms (5,278 in total), each of which appears at least 10 times; these terms are grouped into five clusters. The top 10 most frequent keywords were depression (374), olfaction (159), anxiety (158), dysfunction (123), olfactory bulbectomy (116), olfactory dysfunction (111), PD (100), odor identification (98), brain (96), and disorders (93). The analysis generated five clusters of articles based on their topics, namely, clinical characteristics, pathogenesis, mechanisms, pathological physiology, and the treatment of OD with depression or anxiety. Figure 5B displays the distribution of keywords in chronological order, which is indicated by the depth of the color of the area. Most research before 2012 focused on “clinical characteristics,” whereas the newly identified research hotspots indicate that “mechanism” and “treatment” seem to be the focus of future research. The density of the keywords was based on their frequency and displayed in the form of a density graph (Figure 5C).

CiteSpace identified keywords with citation bursts, which are considered to be indicators of emerging trends. The top 10 keywords with the strongest citation bursts in this field are shown in Figure 5D. The blue line represents the total time span and the burst period is marked by the red line to show the start and end years of the burst. Before 2012, “olfactory bulbectomized rat” had the strongest burst intensity (9.97), whereas the “central nervous system” had the greatest burst strength in 2012–2016, (4.92). After 2016, the keyword with the strongest citation burst was “oxidative stress” (9.63). These changes over time indicate changes in research focus.

### Characteristics of the top 10 most cited articles

The top 10 most cited articles are shown in Table 4, ranked by their number of citations. The number of citations of these articles (7,848) accounted for 18.9% of the total number of all citations (41,480). The article “Mechanisms and functional implications of adult neurogenesis” by Zhao et al. (2008), which was published in *Cell* in 2008 was the most frequently cited article (2,251). Of the top 10 most cited publications, three were published in journals with an impact factor ≥20 (*Cell*; *Lancet Neurology*; and *Nature Reviews Drug Discovery*) and five were published in journals with an impact factor between ≥5 and <20 (*Neuropsychopharmacology*; *Brain Pathology*; *Neuroscience and Biobehavioral Reviews*; *Molecular Psychiatry*; and *Progress in Neuro-Psychopharmacology & Biological Psychiatry*).

**TABLE 4:** Top 10 most co-cited references about OD with depression or anxiety from 2002 to 2021.

Rank	Title	Total citations	First author	Year	Journal
1	Mechanisms and functional implications of adult neurogenesis	2,251	Zhao C. M.	2008	Cell
2	Non-motor symptoms of Parkinson’s disease: diagnosis and management	1,662	Chaudhuri K. R.	2006	Lancet Neurology
3	The ascent of mouse: Advances in modelling human depression and anxiety	850	Cryan J. F.	2005	Nature Reviews Drug Discovery
4	Autism-like behavioral phenotypes in BTBR T+tf/J mice	529	McFarlane H. G.	2008	Genes, Brain and Behavior
5	The olfactory bulbectomised rat as a model of depression	517	Song C.	2005	Neuroscience and Biobehavioral Reviews
6	In search of a depressed mouse: utility of models for studying depression-related behavior in genetically modified mice	443	Cryan J. F.	2004	Molecular Psychiatry
7	The new ‘5-HT’ hypothesis of depression: Cell-mediated immune activation induces indoleamine 2,3-dioxygenase, which leads to lower plasma tryptophan and an increased synthesis of detrimental tryptophan catabolites (TRYCATs), both of which contribute to the onset of depression	425	Maes M.	2011	Progress in Neuro-Psychopharmacology & Biological Psychiatry
8	Olfactory Disorders and Quality of Life–An Updated Review	410	Croy I.	2014	Chemical Senses
9	Profound impairment in social recognition and reduction in anxiety-like behavior in vasopressin V1a receptor knockout mice	382	Bielsky I. F.	2004	Neuropsychopharmacology
10	Mouse behavioral assays relevant to the symptoms of autism	379	Crawley J. N.	2007	Brain Pathology

### Citation analysis

**FIGURE 6:**
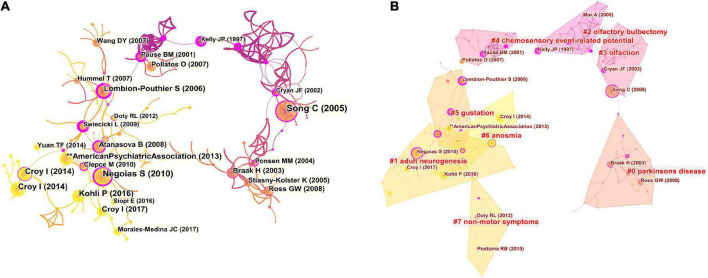
**(A)** The network of co-cited references. **(B)** The network of co-cited reference clusters.

**FIGURE 7:**
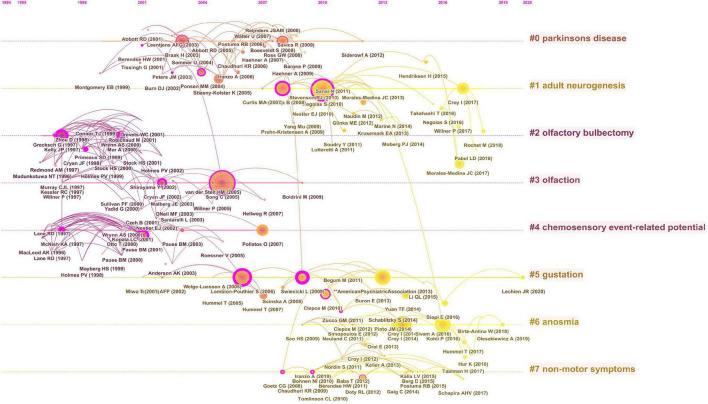
Timeline visualization of the co-cited references map.

**FIGURE 8:**
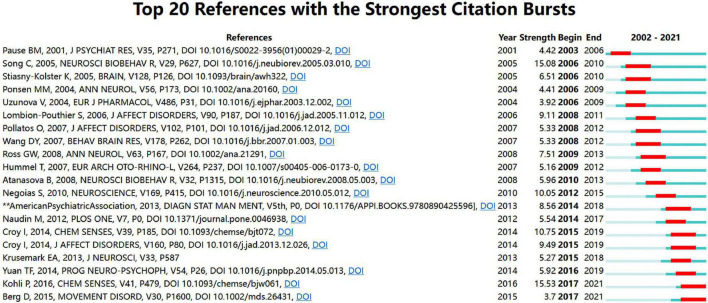
Top 10 references with the strongest citation bursts (2002–2021).

Analysis of cited references is considered to be a crucial part of bibliometric studies. A total of 53,331 cited references from 1,056 articles were included in the co-citation analysis, and a cluster graph was constructed based on their co-occurrence. Figure 6A shows a visual network of co-cited references, which consisted of 219 nodes and 364 links. Each node represents a cited article, with the links between nodes indicating the frequency of being cited for the same reference. The diameter of a node is proportional to the frequency with which the cited reference is quoted. Figure 6B depicts CiteSpace’s clustering of the reference co-citation network, showing only the largest eight clusters, based on index terms and identified by their log-likelihood ratio algorithm. The largest cluster was “PD” (#0), followed by “adult neurogenesis” (#1), “olfactory bulbectomy” (#2), “olfaction” (#3), “chemosensory event-related potential” (#4), “gustation” (#5), “anosmia” (#6), and “non-motor symptoms” (#7). Figure 7 presents the top eight clusters in the form of a timeline that portrays the clusters together with horizontal timelines in descending order, thus indicating the scientific relevance of the references. The top 20 references with the strongest citation bursts are shown in Figure 8, which identifies emerging research hotspots in relevant areas. Most of the bursts are neuroscience-related articles, indicating that neuroscience is a hot subject in OD with depression or anxiety.

### Distribution by journal

Table 5 displays the features of the top 10 most productive journals. These journals accounted for 18.2% (192 publications) of the total number of published articles, and 20% of these journals are in the first quartile (Q1). Among these journals, three have published more than 20 articles. *Behavioural Brain Research*, *PLOS One*, and *Movement Disorders* published the most articles (total = 82 articles, 7.8%), with *Behavioural Brain Research* publishing the largest number of articles (35) and having the largest H-index (17). However, *Movement Disorders* had the highest impact factor and the average number of citations. The Journal Citation Report Q1 contained *Movement Disorders* and *Chemical Senses*; Q2 included *Behavioural Brain Research*, *PLOS One*, *Progress in Neuro-Psychopharmacology & Biological Psychiatry*, *Parkinsonism & Related Disorders*, *Neuropharmacology*, and *Frontiers in Behavioral Neuroscience*, whereas *Brain Research* and *Neuroscience* were ranked as Q3.

**TABLE 5:** Top 10 journals publishing articles on OD with depression or anxiety from 2002 to 2021.

Rank	Journal	Output	Country	Journal citation reports (2021)	Impact factor (2021)	Total cites	Average number of citations	H-index
1	Behavioural Brain Research	35	Netherlands	Q2	3.352	1,113	31.80	17
2	PLOS One	26	United States	Q2	3.752	741	28.50	16
3	Movement Disorders	21	United States	Q1	9.698	1,444	68.76	17
4	Chemical Senses	19	France	Q1	4.985	1,034	54.42	13
5	Brain Research	17	Netherlands	Q3	3.610	506	29.76	10
6	Neuroscience	17	United Kingdom	Q3	3.708	1,013	59.59	13
7	Progress in Neuro-Psychopharmacology & Biological Psychiatry	17	United Kingdom	Q2	5.201	1,286	75.65	13
8	Parkinsonism & Related Disorders	15	United Kingdom	Q2	4.402	752	50.13	13
9	Neuropharmacology	13	United Kingdom	Q2	5.273	587	45.15	12
10	Frontiers in Behavioral Neuroscience	12	Switzerland	Q2	3.617	290	24.17	8

**FIGURE 9:**
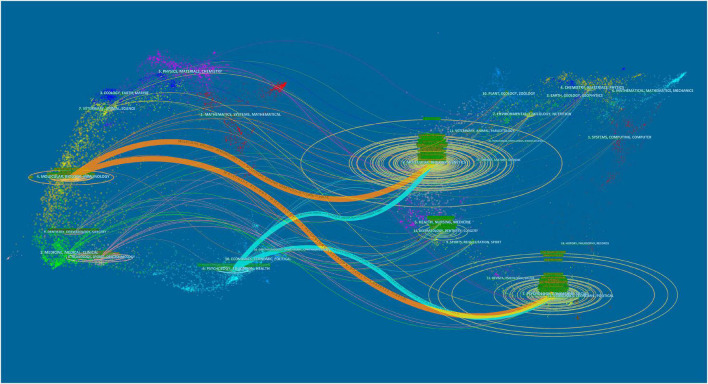
A dual-map overlay of journals.

Figure 9 displays a dual map of journals: the cited journals are on the right side of the figure and where they are cited are on the left side. The distinct lines between citing and cited journals illustrate that the citation links were sourced from different subjects. The names of journals are marked on the vertical axis of the oval. The main citation paths in the dual map are depicted as two orange paths and two green paths. The orange paths indicate articles published in molecular/biology/immunology journals that are cited in molecular/biology/genetics and psychology/education/social journals. The green paths indicate studies published in psychology/education/health journals that are commonly cited in molecular/biology/genetics and psychology/education/sociology journals.

## Discussion

Olfaction is crucial for a broad range of behaviors, such as food localization, emotional regulation, perception of danger, sexual behavior, and social behaviors (Croy et al., 2014; Marin et al., 2018). The olfactory bulbs project to brain regions that play a vital role in emotion and cognition, such as the orbitofrontal cortex, hippocampus, and amygdala (Kohli et al., 2016). A large amount of evidence shows that OD is related to various neuropsychiatric disorders, including depression and anxiety (Fang et al., 2021). Functional and structural damage to the olfactory system, particularly the olfactory bulbs and olfactory epithelium, are common in patients suffering from depression (Zuo et al., 2018). The underlying mechanisms of how OD triggers depressive-like behaviors are complex and need to be examined further.

This article investigated research hotspots and frontiers in OD with depression or anxiety by focusing on the worldwide status and trends in research. Although studies in this area are extensive, they are relatively chaotic and lack analysis of research foci and trends. This is the first time a bibliometric analysis has been conducted to identify emerging trends and hotspots in research on OD with depression or anxiety. Our study found that the research on these topics has increased gradually over the past two decades, with a growing number of studies concentrating on these topics, worldwide.

Our analysis of contributions by country revealed that developed European countries and the United States have dominated this field of research. The United States leads the way by contributing close to 30% of all the publications, which may be related to the size of its population and its financial investment. Significantly, the majority of the top 10 active institutions conducting this research are located in Europe and the United States, which reflects the fact that these countries are well equipped to conduct basic medical science and clinical medicine research because they provide ample funding, have superior equipment, and specialized research staff. Simultaneously, it demonstrates the urgent need in these countries to have effective treatment for OD. Furthermore, the United States has the highest centrality score and is the most active country in international cooperation. It is surprising that Germany, despite being in second place in terms of publications, appears to have less cooperation with other countries and has a lower positive centrality score. The institutions that collaborate most frequently with other institutions are the University of California, Harvard University, and National Institutes of Health. However, the top 10 authors of most publications in this domain all have a centrality of less than 0.01. This suggests that there is insufficient collaboration among top researchers, which may be related to their research being comparatively full-fledged. Overall, cooperation among countries, institutions, and authors is rare. In the future, further collaboration across different levels of research should be conducted to obtain more significant evidence and achieve an enormous breakthrough.

Our analysis of keywords revealed that research on the clinical characteristics, pathophysiology, mechanisms, and treatment of patients with OD and depression or anxiety disorders has attracted extensive attention in the last two decades. Analyzing the keywords with the most burst citations can, to some extent, reveal the development of trends and transformational mutation points in a discipline, and indicate potentially valuable research directions. Our results demonstrate that the interests of scholars were distinct at different stages. During the early phase of research (between 2002 and 2011), academic circles focused greater attention on the clinical characteristics and treatment of OD. Notably, “olfactory bulbectomized rat” is an important keyword with the strongest burst of citations in earlier years. The olfactory bulbectomized rat has been generally acknowledged in academic circles to be an animal model of depression (Morales-Medina et al., 2017). Removal of the olfactory bulbs leads to many abnormalities in neurotransmitter secretion and the endocrine and immune systems in rodents, and the behavioral changes it produces are similar to those observed in patients with depression (Song and Leonard, 2005). Since the behavioral deficits induced in animals with their olfactory bulbs removed are reversed after repetitive antidepressant administration, it is a commonly used model to test the effectiveness of potential antidepressants (Sato et al., 2008). The olfactory bulbectomized model of depression has been confirmed by a range of scientific evidence showing that there is an association between the functions of the olfactory bulbs and depression in humans and rodents (Landis et al., 2012). As time went on, *in vitro* studies have received sustained attention. At a later stage (from 2012 to 2021), the mechanisms underlying the association of OD with depression or anxiety (such as messenger RNA and oxidative stress) have emerged as more prominent research hotspots. Substantial evidence suggests that oxidative stress is an early reaction in neurodegenerative diseases, in which neuronal injury in vulnerable groups of neurons can surpass 90% (Saleem et al., 2020). Studies have revealed that oxidative stress causes oxidative modifications in specific cells of the olfactory bulbs, which is a primary mechanism for age-related OD (Vaishnav et al., 2007).

Journal analysis can provide valuable information that can assist researchers in selecting relevant journals to submit their findings. Our study indicates that neuroscience and psychiatry journals publish the most articles in this domain, which implies that the association of OD with depression or anxiety is one of the core topics of neuroscience and psychiatry. We found that the top 10 most prolific journals published less than one-fifth (18.2%) of the total papers in the area, indicating that the distribution of the literature among journals is significantly dispersed, probably due to the diversity of research directions, which cover neuroscience, psychiatry, clinical neurology, pharmacology, behavioral sciences, and biochemistry-molecular biology. The visualization map in Figure 9 displays the distribution of scientific journals by discipline, as well as the evolution of research content by discipline on a macro level. From the four main paths in the dual map of journals, it is apparent that research on OD with depression or anxiety has been a multidisciplinary effort. Molecular, biology, immunology, psychology, education, and health are the essential and core subjects of OD with depression or anxiety. As the majority of research in this field is interdisciplinary, international collaboration among researchers should be strengthened to yield high-quality research findings.

The timeline of the cluster map of co-cited references shows the high level of interest in the mechanisms of OD accompanying depression in academic circles. A substantial amount of literature suggests a strong link between OD and depression. The cross-sectional study conducted by Seo et al. (2009) on the Korean general population showed that subjects with poorer olfactory function had higher levels of depression and poorer cognitive performance. A recent nationally representative longitudinal study in the United States found that people with olfactory impairment were more likely to experience frequent depressive symptoms in the future compared to healthy people (Eliyan et al., 2020). These data suggest that OD predicts subsequent development of depression in adults and that the olfactory function test may be a useful tool to screen for depression in the general population. Sanna et al. (2021) also found that in addition to olfactory function, other factors such as age, gender, and cognitive function were associated with depression. Conversely, the course and duration of depression can have a dramatic effect on olfactory function (Pabel et al., 2018). In addition, it has been shown that the severity of depression is also closely related to olfactory function. It was reported that odor identification and odor recognition memory were poorer in patients with severe major depressive disorders compared to those with mild major depressive disorders (Zucco and Bollini, 2011).

### Strengths and limitations

This study performed, for the first time, a comprehensive, objective, and visual analysis of publications related to OD with anxiety or depression and their developing trends, which can provide a comprehensive reference for researchers engaged in this topic. In addition, we used various bibliometric software to investigate different dimensions of research hotspots, enabling us to obtain more accurate and objective findings. Inevitably, this study has some limitations. First, the literature included in this study may not be exhaustive. As the WoSCC database is an authoritative, comprehensive, and diverse database that indexes core journal citations, and has been shown to be more accurate than some other databases (especially in the natural sciences (You et al., 2021)), we did not search other databases. Moreover, this study only included original research and reviews, and the language was limited to English, which may have reduced the number of publications retrieved and led to the omission of some publications. The inability of the bibliometric software to distinguish between manuscripts focused on human and animal models is another limitation of this study. Finally, a small number of authors in the data may have duplicate names, and some of the authors may have honorary positions or part-time positions at different universities. Nevertheless, we still believe that this study accurately illustrates the current status and general trends in research on this topic. This study has established a foundation for neuroscientists, otolaryngologists, and related researchers to rapidly identify research foci and emerging research trends in OD with depression or anxiety.

## Conclusion

This is the first study to use bibliometric analysis to provide information about developing trends and foci of research articles related to OD with depression or anxiety during the past two decades. These results may lay a foundation for relevant research in the field worldwide. During the past two decades, the number of relevant articles has grown significantly. In addition, scholars can conduct more relevant research in the field of neuroscience and study the association of OD with neurodegenerative diseases, especially Alzheimer’s disease and PD. Notably, oxidative stress is another hot topic in this field. Simultaneously, relevant researchers may consider publishing more original studies or reviews in psychiatry or neuroscience journals in future. It would be highly valuable to have further exploration of the molecular biological mechanism of OD with depression or anxiety and to determine new effective treatments in later studies. We hope that this work will be instructive for researchers and assist them in improving future research quality, and ultimately benefit patients with OD.

## Data availability statement

The raw data supporting the conclusions of this article will be made available by the authors, without undue reservation.

## Author contributions

FZ and GY conceived the study. FZ, TZ, and YJ collected the data. FZ and YM re-examined the data. YL and MZ analyzed the data. FZ wrote the manuscript. GY and TZ reviewed and revised the manuscript. All authors contributed to the article and approved the submitted version.

## Footnotes

1
http://bibliometric.com/
